# Legal requirements for reporting clinical cases to the South African police or social services

**DOI:** 10.4102/safp.v66i1.5919

**Published:** 2024-06-29

**Authors:** Dirk T. Hagemeister, William Oosthuizen, Bridgette K. Mokae

**Affiliations:** 1 Department of Family Medicine, University of the Free State, Bloemfontein, South Africa; 2 Department of Family Medicine, Free State Department of Health, Bloemfontein, South Africa; 3 Department of Legal Affairs, South African Medical Association, Pretoria, South Africa; 4 Department of Criminology, Faculty of Humanities, University of the Free State, Bloemfontein, South Africa

**Keywords:** mandatory reporting, South Africa, abuse, vulnerable populations, delivery of healthcare

## Abstract

Medical confidentiality is the cornerstone for a trustful relationship between patients and the health professionals attending to them. However, when history or clinical findings suggest certain offenses, statutory laws (*Children’s Act, Older Persons Act, Mental Health Care Act, Sexual Offenses Act*) establish a legal obligation for health professionals to report suspected instances of abuse to the police or alternatively, in some cases, to a designated social worker. Given the high rate of domestic violence and abuse in South Africa, health professionals are most likely to encounter such situations. Many clinicians are oblivious of the obligations, exposing themselves to possible liability and their patients to potential additional harm. This article aims to demonstrate the reporting requirements under the respective acts through case scenarios. Finally, the advantages and disadvantages of the existing legal setting are discussed briefly.

## Introduction

Open and trustful communication between patient and clinician is the foundation of any clinical consultation. Anyone seeking assistance needs to share the nature of the health concern, whether it is physical or mental. Professional ethics and legislation protect the therapeutic relationship. The protection of medical confidentiality dates back to times immemorial as evidenced in the Hippocratic Oath.^1^ Centuries later, the booklet by the Health Professions Council of South Africa (HPCSA) provides guidance in this regard.^2^ Today, the protection of confidential information is regarded as a realisation of the bioethical principles of respect for the patient’s autonomy.^3^

Section 14 of the *National Health Act (NHA)* codifies the confidentiality of all information shared by the patient and additional data gained by examination or investigation during the healthcare encounter^4^:

‘14. Confidentiality

1. All information concerning a user, including information relating to his or her health status, treatment or stay in a health establishment, is confidential.
2. Subject to section 15, no person may disclose any information contemplated in subsection (1) unless –
  - the user consents to that disclosure in writing
  - a court order or any law requires that disclosure
  - nondisclosure of the information represents a serious threat to public health’.

The disclosure as contemplated by subsection 2(b) might be required for the processing of claims, the prevention of health threats to the community or the protection of vulnerable individuals. Neither billing between healthcare providers and funders nor infectious disease outbreaks requiring reporting to the authorities, are discussed here. Our focus is exclusively on vulnerable individuals where the clinician notices evidence of certain transgressions during the consultation. Whether legally incapacitated by old age or mental illness, or whether not yet legally competent because of young age, vulnerable individuals enjoy special protection by the state’s authorities and are therefore subject to such reporting duties. In particular, the *Children’s Act*, Act 38 of 2005^5^ bears reference where child abuse or neglect is of concern, and the *Older Persons Act*, Act 13 of 2006^6^ similarly provides protection for older people. In the case of mental incapacity, the *Mental Health Care Act*, Act 17 of 2002^7^ determines processes to be followed to protect this vulnerable group from harm, and the Criminal Law (Sexual Offences and Related Matters) *Amendment Act* (CLSOAA), Act 32 of 2007^8^ sets out, among other, to prevent sexual abuse or exploitation of a range of vulnerable groups. The *Domestic Violence Act*, Act 116 of 1998^9^ finally offers legal protection for a list of groups, including a child, a person with a disability or an older person, from domestic violence. Interestingly, the standard of proof that triggers the clinician’s duty to report ranges from mere suspicion in some acts to conclusion on reasonable grounds in others.

In the following vignettes, clinical scenarios are placed in context of the relevant legislation. Resulting reporting duties for each of the cases are presented.

## Case vignettes

### Case 1

You are called to assist with the care of a 2-year-old boy with second-degree burns of both buttocks. The 24-year-old mother, appearing intoxicated, claims to have been heating water for the bath and that the child then placed himself into the boiling hot water. The child is in acute pain, but even after adequate pain medication, appears withdrawn.

#### Clinical considerations

Initial clinical care of this child includes analgesia, wound care, fluid management, thorough examination and where available, referral for specialised burn care.

#### Legal aspects

Presentation and history given by the parent cause suspicion. Section 7 of the *Children’s Act*^5^ aims to realise the Best Interests of Child Standard mandated by section 28(2) of the constitution.^10^ Section 110 of the same act^5^ provides a long list of health and other professionals who deal with children and obliges that any of them:

[*W*]ho on reasonable grounds concludes that a child has been abused in a manner causing physical injury, sexually abused or deliberately neglected, must report that conclusion in the prescribed form to a designated child protection organisation, the provincial department of social development or a police official.

This prescribed form is a form 22 from the relevant regulations, and the remainder of section 110 deals with the prescribed interactions between the three stakeholders involved, that is, a child protection organisation, the Department of Social Development and the police. These processes are aimed at protecting the child from further harm, including separation of the child from the alleged perpetrator.

Section 2A of the *Domestic Violence Act* mandates that:

A functionary, who in the course of the performance of their duties or the exercise of their functions obtains information which, after evaluation by them, causes them to believe or suspect on reasonable grounds, that a child, […] may be a complainant […] must without delay complete a report in the prescribed form.^9^

#### Requirements

The attending clinician, based on *conclusion on reasonable grounds*, legally has no choice but to report to any of the three authorities mentioned in the *Childrens Act*, failing of which the professional might be found guilty of an offence (section 305) and be sentenced to a fine or imprisonment of up to 10 years.^5^ (The common practice seen that the hospital-employed social worker gets involved with such cases, is a means to achieve the above. However, such social worker would still have the legal obligation to report in the prescribed manner, and failing to do so, especially if further damage occurs to the child, might result in criminal liability on the side of the clinician). A consideration of this case under the *Domestic Violence Act*, based on *belief or suspicion on reasonable grounds*, would result in a similar conclusion.^9^ Both acts independently also introduce mechanisms to ensure the safety of the identified victim that go beyond the mere duty to report, which in section 2A(2)(b) prescribes conducting a risk assessment by the functionary (clinician) involved.

### Case 2

A 13-year-old girl, accompanied by her mother, is requesting a termination of her pregnancy. She is unsure about the date of her last menstruation, and the ultrasound examination shows an intrauterine singleton pregnancy estimated at 12 weeks and 6 days.

#### Clinical considerations

No immediate medical intervention is necessary. Gestational age estimations are important in the legal context^11^ and relevant clinical protocols need to be followed.^12^

#### Legal aspects

The *Choice on Termination of Pregnancy Act (CToPA)* (section 1) enables any woman to request a termination of pregnancy, without specifying a minimum age.^13^ In the second trimester, that is, after completing 12 weeks of pregnancy, a medical practitioner needs to confirm the indication. Unlike surgical procedures in the *Children’s Act* (5) (section 129(3)), parental consent or involvement is not required by the CToPA. The medical practitioner needs to consider that the age of consent for sexual intercourse in South Africa is 16 years. The *Criminal Law (Sexual Offences and Related Matters) Amendment Act (CLSOAA)*^8^ stipulates that children between 12 and 16 years of age may consent to sexual activities if the partner is in the same age bracket, or if older than 16, not more than 2 years older than the child (section 15). Any child younger than 12 years is legally incapable of consenting (section 57). Any intercourse, consensual or not, with a child younger than 12 years, or intercourse with a child between 12 and 16 years by a person older than stipulated in section 15, constitutes rape. Section 54 prescribes that: [a] person who has knowledge, reasonable belief or suspicion that a sexual offence has been committed against a person who is vulnerable […] must report […] immediately to a police official.^14^

#### Requirements

Legally, the age of the biological father of the foetus is important. It remains debatable whether the attending clinician has to inquire about the father’s age to establish whether or not a statutory rape was committed. However, if the facts are established and documented, such statutory rape has to be reported to the police, bearing in mind that the Act also refers to mere suspicion. (From a forensic point of view, it might be important to consider that the products of conception constitute genetically determined material and as such might serve as evidence in criminal proceedings).

### Case 3

A 25-year-old mental healthcare user with severe intellectual impairment, living in a chronic care facility, is brought in because her ‘tummy is swollen’. On examination, the gravid uterus has a symphysis-fundus height (SFH) of 28 cm and foetal movements are palpable. The staff at the chronic care facility is not aware of any ‘relationship’, and no family members of the patient are on record.

#### Clinical considerations

Again, this is not an emergency. Biologically, the 25-year-old might experience a low-risk pregnancy, leaving the main concern with the social and emotional consequences.

#### Legal aspects

An institutionalised mental healthcare user enjoys special protection under South African law. (Decisions about the possible termination of the pregnancy under section 5(4) of the CToPA, which at an advanced stage might not be legally possible anymore, or the ethical, social and legal deliberations regarding the upbringing and care of the child, would possibly warrant a separate article and have not been considered here). Firstly, section 11 of the *Mental Health Care Act* protects the user against exploitation or abuse^7^ and establishes a legal duty to report the incident to the Mental Health Review Board or the SA Police Services.^15^ Section 14, however, makes the not entirely helpful statement that:

[*T*]he head of a health establishment may limit intimate relationships of adult mental health care users only if due to mental illness, the ability of the user to consent is diminished.

Thus leaving the onus with the said head of the establishment. Secondly, the CLSOAA,^8^ section 57 dealing with children under the age of 12, also states categorically that: ‘a person with a mental disability is incapable of consenting to a sexual act’, and defines in section 1 of the same act such mental disability as being ‘unable to appreciate the nature and consequences of a sexual act’, being ‘unable to act in accordance with that appreciation’, ‘unable to resist’ or ‘unable to communicate […] unwillingness’. Dealing again with a vulnerable person as defined in section 40 of the act, the obligation to report to a police official immediately (section 54), *even on mere suspicion*, also applies in this case.

#### Requirements

Although this is a complex case with a plethora of ethical and social questions, there is a clearly defined legal reporting duty. The relevant authorities will then have to investigate the matter.

### Case 4

A 68-year-old male resident of a retirement home is brought to the hospital because of a reduced level of consciousness. Having been diagnosed previously with multi-infarct dementia, despite independently mobilising out of bed, he is care-dependent. His back shows two sets of parallel contusion marks, highly suggestive of physical assault.

#### Clinical considerations

This older patient might well be vitally threatened and will require a focused clinical work-up assessing for possible additional injuries or even a crush injury threating to the kidneys.

#### Legal aspects

In South Africa, section 1 of the *Older Persons Act*^6^ defines an older person as a female from the age of 60 years and a male from the age of 65 years. Similar to the other cases above, the *Older Persons Act* puts legal protection for older persons as a vulnerable group in place, including reporting obligations. Section 25 prescribes that ‘(a)ny person who is involved with an older person in a professional capacity’ must report identified need of an older person for care or protection to the department of social development. Section 26 throws the net even wider when it obliges ‘[*a*]ny person who suspects that an older person has been abused […] must immediately notify the Director-General [*of social development*] or a police official of his or her suspicion’.^6^

#### Requirements

Clinicians who encounter suspected physical abuse of an older person legally have no choice but to report it, based on as little as a suspicion. It is no longer the affected older person’s decision whether to open a case of assault. Similar to children or mentally impaired people, as soon as they confide in a person attending to them professionally, such a report is required.

### Case 5

A 23-year-old female medical student asks you in confidence to assist her with a morning-after pill. She shares that she stays at the student residence, that she was quite intoxicated over the weekend and does not have full recollection of what happened. However, she has good reason to believe that one of her male fellow residents had intercourse with her while she was intoxicated.

#### Clinical considerations

This case appears uncomplicated, both medically and legally. Advice about post-exposure prophylaxis and the requested contraceptive and its prescription appear adequate for a competent adult.

#### Legal aspects

The 2022 amendment^16^ of the CLSOAA^8^ introduced a definition of *a person who is vulnerable*, including a female under the age of 25 who receives tuition at a higher education institution (section 40, amendment of Act 32 of 2007). The definition of consent in section 1(3) determines that ‘under the influence of alcohol to an extent that the consciousness is affected’, a person is incapable of giving consent. Without valid consent, sexual penetration constitutes rape, which is one of the sexual offences that have to be reported if committed on a vulnerable person (section 54).^8^

#### Requirements

The medical practitioner finds himself or herself in the uncomfortable position of committing a punishable offence if not reporting the suspicion to the police immediately, despite the student who is contacting the practitioner in confidence has no intention of doing so.

## Discussion and summary

It is key to differentiate between victims of assault who are expected by the law to make their own decision, whether to open a criminal case or seek protection from the authorities against the alleged perpetrator, and those population groups for which the law, in a paternalistic way, takes legal stewardship for the victim and mandates professionals to report.

Table 1 provides a summary of vulnerable groups, offences, reporting practices, applicable acts and the consequences of failure to comply. Suspected abuse often happens in a complex domestic context of care provision and dependency. The clinician then becomes another player in the challenging social interaction and needs to carefully consider the trust-based relationship to the victim (and possibly the perpetrator). Sometimes the dedicated clinician might feel that reporting is not in the patient’s best interest (the guiding principle of the *Children’s Act*), or that the reporting duty in one piece of legislation (such as the CLSOAA) directly contradicts other pieces of legislation (the CToPA) and might negatively affect health outcomes by changing health-seeking behaviour. In the Teddy Bear Clinic case, the Constitutional Court stated clearly that the punitive code of law was not the appropriate tool to ensure sex education of teenagers, subsequently instructing the legislature to amend the then CLSOAA.^17^

**TABLE 1:** Summary of vulnerable groups, offenses, reporting, applicable acts and consequences of failure to comply.

Vulnerable group	Offence to report	Minimum standard of proof	Report to	Prescribed form	Who has to report	Applicable act	Punishable if failure to comply
Children	Abuse causing physical injury, sexual abuse, deliberate neglect	Conclusion on reasonable grounds	Designated child protection organisation, or Provincial Department of Social Development (DSD), or Police official	Form 22, GG 33076	Any correctional official, dentist, homeopath, immigration official, labour inspector, legal practitioner, medical practitioner, midwife, minister of religion, nurse, occupational therapist, physiotherapist, psychologist, religious leader, social service professional, social worker, speech therapist, teacher, traditional health practitioner, traditional leader or member of staff or volunteer worker at a partial care facility, drop-in centre or child and youth care centre	*Children’s Act*, section 110	Yes; fine and/or up to 10 years in prison (section 305)
	(Statutory) rape	Suspicion	Police official	None	A person who has knowledge, reasonable belief or suspicion	*Sexual Offences Act*, sections 15 & 57	Yes; fine and/or up to 5 years in prison (section 54(2))
Mental healthcare users	Any form of abuse against a mental healthcare user	Own observation	Institution’s review board, or Police official	MHCA Form 02 or lay charge with the police	A person witnessing the incident	*Mental Health Care Act*, section 11; regulation 7	No; failure to report is not listed as an offence in section 70
Child, a person with disability or older person	Act of violence (as defined) by person in domestic relationship	Belief or suspicion on reasonable grounds, evaluation of information	Social worker or Member of the South African Police Service	Form 2, Regulation 4(1), GG48428	A functionary as defined in the act, explicitly including medical practitioners	*Domestic Violence Act*, section 2A	No; offence only defined if not a functionary
Vulnerable persons	Sexual offence committed against a person who is vulnerable	suspicion	Police official	None	A person who has knowledge, reasonable belief or suspicion	*Sexual Offenses Act*, section 40 (definition); section 54	Yes; fine and/or up to 5 years in prison (section 54(2))
Older persons Male ≥ 65 years, Female ≥ 60 years	Need for care or protection	Conclusion on personal observation	Director-General (DG) of the DSD	None	Any person who is involved with an older person in a professional capacity	*Older Persons Act*, section 25	No
	Abuse	Suspicion	DG of the DSD, or Police official	None	***Any person*** who suspects that an older person has been abused or suffers from an abuse related injury	*Older Persons Act*, section 26	Yes; fine and/or up to 5 years in prison (section 33(b))

MHCA, *Mental Health Care Act*.

While there certainly is no space here to honour the need for a full ethical discussion of the matters, no vulnerable patients should be harmed by a clinician’s legal oblivion. At the same time, the seasoned clinician will be aware that some crucial information regarding potential transgressions that is willingly shared with the clinician, might not be as easily volunteered to the police or social development authorities.
